# Antibiotic Treatment in Patients Hospitalized for Nonsevere COVID-19

**DOI:** 10.1001/jamanetworkopen.2025.11499

**Published:** 2025-05-19

**Authors:** Michael S. Pulia, Meggie Griffin, Rebecca Schwei, Aurora Pop-Vicas, Lucas T. Schulz, Meng-Shiou Shieh, Penelope Pekow, Valerie M. Vaughn, Peter K. Lindenauer

**Affiliations:** 1 BerbeeWalsh Department of Emergency Medicine, University of Wisconsin-Madison School of Medicine and Public Health, Madison; 2 Department of Industrial and Systems Engineering, University of Wisconsin-Madison, Madison; 3 Division of Infectious Diseases, Department of Medicine, University of Wisconsin-Madison School of Medicine and Public Health, Madison; 4 School of Pharmacy, University of Wisconsin-Madison, Madison; 5 Department Healthcare Delivery and Population Sciences, University of Massachusetts Chan Medical School-Baystate, Springfield; 6 Division of General Internal Medicine, Department of Internal Medicine, University of Utah School of Medicine, Salt Lake City; 7 Division of Health System Innovation & Research, Department of Population Health Sciences, University of Utah School of Medicine, Salt Lake City; 8 Department of Medicine, University of Massachusetts Chan Medical School-Baystate, Springfield

## Abstract

**Importance:**

Patients hospitalized with nonsevere COVID-19 continue to receive community-acquired pneumonia (CAP) antibiotic treatment despite a low risk of bacterial coinfection. Unnecessary antibiotic prescribing contributes to global antibiotic resistance and also poses a threat to individual patients.

**Objective:**

To examine the association of CAP antibiotic treatment started on admission with clinical outcomes among a large sample of patients hospitalized for nonsevere COVID-19 in hospitals across the US.

**Design, Setting, and Participants:**

This retrospective cohort study used a target trial emulation design. Participants were adult, immunocompetent patients admitted to general care for COVID-19 from April 2020 to December 2023 at 1053 US-based acute-care hospitals that contribute data to the Premier Healthcare Database. Patients with nonpneumonia bacterial infections present on admission were excluded. Data were analyzed from April to October 2024.

**Exposure:**

Receipt of a CAP antibiotic regimen on the day of admission.

**Main Outcomes and Measures:**

The primary outcome was a composite measure of deterioration (vasopressor, high-flow oxygen, noninvasive ventilation, invasive mechanical ventilation, intermediate care, intensive care unit admission) and in-hospital mortality occurring on day 2 or later. The association between receipt of antibiotic therapy and the primary outcome was assessed using propensity methods while adjusting for a broad set of potential confounders, including cotreatments.

**Results:**

The cohort included 520 405 patients with COVID-19 (median [IQR] age, 66 [53-78] years; 266 186 [51.2%] male), including 92 708 Black patients (17.8%), 63 619 Hispanic patients (12.2%), and 304 649 White patients (58.5%); 279 656 patients (53.7%) had Medicare insurance. A total of 160 482 patients (30.8%) were treated with a CAP antibiotic regimen on day 1 of admission. The primary composite outcome was higher in the CAP group (20.8%) compared with the unexposed (no antibiotic) group (18.4%), but the difference did not meet the predefined criteria for clinical significance (ASD, 4.1%). Patients who received CAP antibiotics had higher odds of poor clinical outcomes (propensity matched–odds ratio [OR], 1.03 [95% CI, 1.01-1.05]; *P* = .003; inverse probability treatment weighted–OR, 1.03 [95% CI, 1.02-1.05]; *P* < .001; standardized mortality ratio weighted–OR, 1.10 [95% CI, 1.08-1.12]; *P* < .001).

**Conclusions and Relevance:**

In this large cohort study of patients hospitalized with nonsevere COVID-19, there was no clinically meaningful difference in outcomes with early antibiotic treatment. Given the risks associated with unnecessary antibiotic treatment, these results argue against routine antibiotic use in this population.

## Key Points

**Question:**

Are antibiotics targeting community-acquired bacterial pneumonia associated with clinical benefit to hospitalized patients as part of treatment for nonsevere COVID-19?

**Findings:**

This cohort study with target trial emulation including 520 405 patients did not find clinically significant absolute differences but did find statically significantly higher odds of clinical deterioration and in-hospital mortality associated with antibiotic treatment for COVID-19 using propensity score–weighted and matched models.

**Meaning:**

This large cohort study found no benefit associated with antibiotic treatment for patients hospitalized with nonsevere COVID-19, thus arguing against its routine use, given the known harms of unnecessary antibiotic treatment.

## Teaser

This cohort study using a target trial emulation design examines the association of community-acquired pneumonia antibiotic treatment started on admission with clinical outcomes among patients hospitalized for nonsevere COVID-19.

## Introduction

Reports consistently demonstrate that inpatients with COVID-19 receive antibiotics at rates higher (>30%) than the approximately 5% of cases involving confirmed bacterial coinfections.^1,2,3^ Clinician concerns for bacterial pneumonia coinfection are thought to drive these antibiotic prescribing rates.^4^ High utilization of antibiotics increases the prevalence of resistant bacterial infections, and in the US alone, 2.8 million antibiotic-resistant infections occur each year, with 35 000 associated deaths.^5,6,7,8,9,10^ Unnecessary antibiotic prescribing also poses a threat to individual patients due to the risk of serious adverse drug events and *Clostridioides difficile* colitis.^5,11^ The clinical benefit of antibiotic treatment for patients hospitalized with nonsevere COVID-19 needs to be assessed due to the high rates of antibiotic prescribing in this population.

Meta-analyses of randomized clinical trials have found azithromycin to be an ineffective treatment for COVID-19.^12,13,14,15^ However, few studies have investigated clinical outcomes beyond mortality for patients with COVID-19 treated with other antibiotics.^16,17,18,19^ Target trial emulation,^20^ the application of a randomized clinical trial framework to reduce bias in observational studies, has been used in several studies to assess treatments for COVID-19.^21,22,23,24,25,26^ We designed a target trial emulation study to examine the association of community-acquired pneumonia (CAP) antibiotic treatment started on admission with clinical deterioration and in-hospital mortality among a large sample of patients hospitalized in the US for nonsevere COVID-19. We hypothesized that there would be no difference in outcomes for patients with COVID-19 treated with a CAP antibiotic regimen compared with those not treated with antibiotics early in their admission.

## Methods

For this cohort study, the University of Wisconsin-Madison institutional review board determined that because all data were fully deidentified this study was not human participants research and therefore was exempt from approval and informed consent. This report follows the Strengthening the Reporting of Observational Studies in Epidemiology (STROBE) reporting guideline for cohort studies.

### Eligibility Criteria

In our emulation of a hypothetical target trial, we used data from the Premier Healthcare Database to identify encounters for COVID-19 from April 2020 to December 2023. The Premier Healthcare Database contains deidentified data for approximately 25% of annual US inpatient admissions compiled from a geographically and structurally diverse group of acute care hospitals.^27^

We included patients with an *International Statistical Classification of Diseases and Related Health Problems, Tenth Revision* (*ICD-10*) code for COVID-19 (U07.1) present on admission (POA) or an *ICD-10* code for COVID-19 not POA and a billing charge for a COVID-19 test on day 1. Adult (age ≥18 years) patients with inpatient or observation encounters for COVID-19 were eligible. To focus on patients with respiratory symptoms, we required that chest imaging occurred on day 1. We excluded patients presenting with nonpneumonia bacterial infections or a chronic obstructive pulmonary disease exacerbation (*ICD-10* code: J44.0/J44.1) because these patients have a separate indication for antibiotic treatment. We excluded patients presenting with nonpneumonia bacterial infections using a tiered antibiotic appropriateness *ICD-10* code framework originally published by Centers for Disease Control and Prevention investigators that classifies diagnoses as always (eg, urinary tract infection), sometimes (eg, acute sinusitis), or never (eg, acute upper respiratory infection) appropriate (eTable 1 in Supplement 1).^28,29,30^ We excluded patients with POA diagnoses where antibiotics are always appropriate and those treated on day 1 with antibiotic regimens not targeting nonsevere CAP (eTable 2 in Supplement 1), as this suggests a suspected nonpneumonia bacterial infection or sepsis concern. Due to differences in antibiotic treatment protocols for patients with immunosuppression, including prophylaxis and broader spectrum coverage, we excluded patients receiving immunomodulating medications (eTable 2 in Supplement 1) on day 1 and patients with neutropenia POA (*ICD-10* code: D70). Finally, we excluded patients who had already reached the primary outcome (eg, admitted to the intensive care unit [ICU]) on the day of admission according to billing and *International Classification of Diseases, Tenth Edition, Procedure Coding System* (*ICD-10-PCS*) procedure codes (eTable 3 and eTable 4 in Supplement 2) and those with unknown sex. The number of patients evaluated at each step of eligibility and assigned to each treatment group are presented in the Figure.

**Figure.**
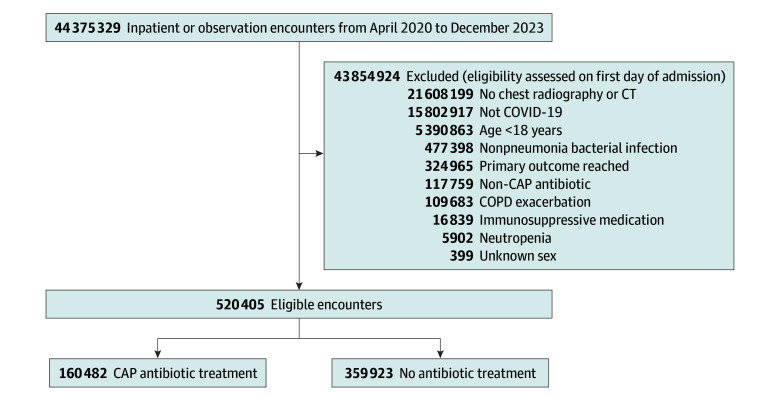
Target Trial Emulation Study Diagram CAP indicates community-acquired pneumonia; COPD, chronic obstructive pulmonary disease; and CT, computed tomography.

### Target Trial Hypothetical Treatment Regimen

Our hypothetical treatment regimen randomized patients to receive a CAP antibiotic regimen or no antibiotics on day 1. We defined a CAP antibiotic regimen as receiving 1 or more of the antibiotic agents listed in the Infectious Diseases Society of America CAP Clinical Pathway^31^: ampicillin/sulbactam, ceftriaxone, cefotaxime, azithromycin, clarithromycin, doxycycline, levofloxacin, and/or moxifloxacin. We defined the exposure for our emulated target trial, ie, the hypothetical treatment regimen, according to billing for CAP antibiotic agents initiated in the emergency department or day 1 of admission. We used an intention-to-treat approach, and treatment group assignment remained the same during analysis regardless of changes in antibiotic therapy, ie, discontinuation or intensification. For example, a patient receiving a single dose of a CAP antibiotic in the emergency department and no further antibiotics inpatient would be analyzed in the exposed group. To assess treatment after day 1, we calculated the percentage of patients who received CAP antibiotic treatment on day 2 or later for both groups and calculated the median days of therapy (DOT; number of unique days of treatment per antibiotic agent).

### Outcomes

Our primary outcome was a composite measure of deterioration (defined as transfer to ICU, intermediate care; step down level of care between ICU and general ward], initiation of vasopressors, high-flow oxygen [heated and humidified oxygen delivered at higher rates than standard nasal cannula], noninvasive ventilation, or invasive mechanical ventilation) and in-hospital mortality occurring on day 2 or later. We assessed deterioration via administrative billing codes and *ICD-10* procedure codes (eTable 3 in Supplement 2). Antibiotic adverse events during the index admission, including kidney failure^32,33^ not POA, allergic reaction,^34^ or *C difficile* infection,^35^ were evaluated as safety outcomes and identified using *ICD-10* codes. All-cause readmission at 30 days (to the same institution) and hospital length of stay were included as secondary outcomes.

### Patient Characteristics and Confounders

Because patients were not randomized to the CAP antibiotic treatment regimen and physicians decided treatment according to patients’ clinical presentation, we compiled data on a variety of potentially confounding factors. We incorporated patient characteristic data, including age, sex (female, male), race and ethnicity (Black, Hispanic, non-Hispanic White, other), and primary insurance payer (Medicaid, Medicare, private, uninsured/other/unknown). Race and ethnicity designations were collected from the standard UB-04 claim form and the race categories American Indian or Alaskan Native and Native Hawaiian or other Pacific Islander were collapsed by Premier into other race and ethnicity to ensure deidentification. Hospital characteristics included number of beds (<200, 200-399, ≥400), census region (Midwest, Northeast, South, West), rurality (urban, rural), and teaching status (yes, no).

Additionally, we identified Elixhauser comorbidities using software (version 2024.1; Healthcare Cost and Utilization Project)^36^ and identified organ failure POA (respiratory, cardiovascular, renal, hepatic, acidosis, neurologic, hematological) based on *ICD-10* codes.^32,33^ We identified COVID-19 treatments (remdesivir, monoclonal antibodies, systemic steroids [hydrocortisone, cortisone, prednisone, prednisolone, methylprednisolone, dexamethasone, betamethasone]) and diagnostic tests (procalcitonin, blood culture, lactate) ordered on day 1. Finally, we generated indicator variables corresponding to each quarter (eg, April-June 2020, July-September 2020) to adjust for evolving SARS-COV-2 variants and treatment over time. Because of our large sample size, we used absolute standardized differences (ASDs), the absolute difference in means divided by the pooled SD, to compare characteristics of the groups.^37^ We considered an ASD greater than 10% to demonstrate clinically meaningful differences between the groups.^38,39^

### Statistical Analysis

We first compared the primary, secondary, and safety outcomes using ASDs for COVID-19 patients that received CAP vs no antibiotic treatment on day 1. We also evaluated the primary outcome by year for each exposure group (eTable 5 in Supplement 2) to describe differences in outcomes over time. Then using methods similar to previous work,^40^ we constructed a series of multivariable models to evaluate the primary outcome by exposure, and in all models, we used generalized estimating equations to account for hospital-level clustering. To calculate propensity scores for use in matching and weighting, we developed a propensity score model to predict CAP antibiotic treatment with patient characteristics, hospital characteristics, individual Elixhauser comorbidities, organ failure, quarter of admission, and day 1 diagnosis and treatment. We then matched patients treated with CAP antibiotics to untreated patients by propensity score (ie, the conditional probability of receiving treatment) using a greedy matching algorithm and conducted conditional logistic regression on the matched pairs. To gauge the effectiveness of propensity matching at balancing the covariates, Table 1 includes ASD comparisons for the matched cohort. Additionally, to explore a range of potential effect estimates, we used weighted propensity score methods, including standardized mortality ratio weighting (SMRW) and inverse probability treatment weighting (IPTW). These methods reweight the entire study cohort based on propensity scores: SMRW estimates the average treatment effect on the treated, while IPTW estimates the average treatment effect if applied to the entire population.^41^ As a sensitivity analysis, we replicated all models for deterioration and in-hospital mortality as separate outcomes (eTable 6 in Supplement 2).

**Table 1.** Characteristics of Patients Hospitalized With COVID-19 Treated With and Without CAP Antibiotics

Characteristics	Unmatched cohorts				Matched cohorts		
	Patients, No. (%)			ASD, %	Patient, No. %		ASD, %
	All (n = 520 405)	No day 1 antibiotic (n = 359 923)	CAP antibiotic day 1 (n = 160 482)		No day 1 antibiotic (n = 113 506)	CAP antibiotic day 1 (n = 113 506)	
Age, median (IQR), y	66 (53-78)	67 (53-78)	65 (52-77)	5.1	66 (53-78)	66 (53-78)	0.3
Sex							
Female	254 219 (48.9)	177 812 (49.4)	76 407 (47.6)	3.6	55 199 (48.6)	54 956 (48.4)	0.4
Male	266 186 (51.2)	182 111 (50.6)	84 075 (52.4)		58 307 (51.4)	58 550 (51.5)	
Race and ethnicity							
Black	92 708 (17.8)	66 139 (18.4)	26 569 (16.6)	10.1	19 141 (16.9)	19 291 (17.0)	0.0
Hispanic	63 619 (12.2)	40 767 (11.3)	22 852 (14.2)		13 943 (12.3)	13 986 (12.3)	
Other^a^	59 429 (11.4)	39 890 (11.1)	19 539 (12.2)		13 461 (11.9)	13 476 (11.9)	
White	304 649 (58.5)	213 127 (59.2)	91 522 (57.0)		66 961 (59.0)	66 753 (58.8)	
Insurance							
Medicaid	70 158 (13.5)	49 255 (13.7)	20 903 (13.0)	8.5	14 605 (12.9)	14 550 (12.8)	0.0
Medicare	279 656 (53.7)	197 410 (54.9)	82 246 (51.3)		61 272 (54.0)	60 953 (53.7)	
Private	128 757 (24.7)	85 890 (23.9)	42 867 (26.7)		28 361 (25.0)	28 635 (25.2)	
Uninsured/other/unknown	41 834 (8.0)	27 368 (7.6)	14 466 (9.0)		9268 (8.2)	9368 (8.3)	
Elixhauser comorbidities, median (IQR), No.	3 (1-4)	3 (1-4)	2 (1-4)	8.6	3 (1-4)	3 (1-4)	0.2
Organ failure, POA							
Acidosis	29 887 (5.7)	18 625 (5.2)	11 262 (7.0)	7.7	7368 (6.5)	7299 (6.4)	0.3
Cardiovascular	38 758 (7.5)	27 299 (7.6)	11 459 (7.1)	1.7	8419 (7.4)	8344 (7.4)	0.3
Hematological	39 431 (7.6)	26 907 (7.5)	12 524 (7.8)	1.2	8902 (7.8)	8941 (7.9)	0.1
Hepatic	3577 (0.7)	2364 (0.7)	1213 (0.8)	1.2	833 (0.7)	817 (0.7)	0.2
Neurologic	43 499 (8.4)	29 895 (8.3)	13 604 (8.5)	0.6	10 344 (9.1)	10 171 (9.0)	0.5
Respiratory	14 916 (2.9)	8785 (2.4)	6131 (3.8)	7.9	3816 (3.4)	3845 (3.4)	0.1
Kidney	107 515 (20.7)	73 515 (20.4)	34 000 (21.2)	1.9	24 484 (21.6)	24 287 (21.4)	0.4
Venous thromboembolism POA							
Deep vein thrombosis	6044 (1.2)	4343 (1.2)	1701 (1.1)	1.4	1304 (1.2)	1294 (1.1)	0.1
Pulmonary embolism	10 422 (2.0)	6977 (1.9)	3445 (2.2)	1.5	2386 (2.1)	2433 (2.1)	0.3
Day 1 diagnostic tests							
Blood culture	197 604 (38.0)	94 310 (26.2)	103 294 (64.4)	83.0	62 023 (54.6)	60 745 (53.5)	2.3
Lactate	307 516 (59.1)	185 902 (51.7)	121 614 (75.8)	51.8	79 972 (70.5)	79 539 (70.1)	0.8
Procalcitonin	190 746 (36.7)	112 354 (31.2)	78 392 (48.9)	36.6	51 477 (45.4)	51 051 (45.0)	0.8
Day 1 COVID-19 treatment							
Monoclonal antibodies	6012 (1.2)	4645 (1.3)	1367 (0.9)	4.3	1100 (1.0)	1107 (1.0)	0.1
Remdesivir	105 029 (20.2)	65 045 (18.1)	39 984 (24.9)	16.7	25 931 (22.9)	25 863 (22.8)	0.1
Systemic steroids	133 046 (25.6)	73 931 (20.5)	59 115 (36.8)	36.6	33 782 (29.8)	33 619 (29.6)	0.3
Hospital bed size, No.							
<200	126 235 (24.3)	81 150 (22.6)	45 085 (28.1)	17.1	29 478 (26.0)	29 729 (26.2)	0.0
200-399	188 379 (36.2)	126 894 (35.3)	61 485 (38.3)		43 126 (38.0)	42 945 (37.8)	
≥400	205 791 (39.5)	151 879 (42.2)	53 912 (33.6)		40 902 (36.0)	40 832 (36.0)	
Hospital region							
Midwest	94 593 (18.2)	68 808 (19.1)	25 785 (16.1)	25.7	19 977 (17.6)	20 027 (17.6)	0.0
Northeast	114 272 (22.0)	88 109 (24.5)	26 163 (16.3)		22 537 (19.9)	22 228 (19.6)	
South	238 359 (45.8)	159 194 (44.2)	79 165 (49.3)		54 202 (47.8)	54 478 (48.0)	
West	73 181 (14.1)	43 812 (12.2)	29 369 (18.3)		16 790 (14.8)	16 773 (14.8)	
Rural hospital	62 961 (12.1)	39 327 (10.9)	23 634 (14.7)	11.4	15 654 (13.8)	15 738 (13.9)	0.2
Teaching hospital	247 399 (47.5)	184 605 (51.3)	62 794 (39.1)	24.6	49 684 (43.8)	50 006 (44.1)	0.6

Abbreviations: ASD, absolute standardized difference; CAP, community-acquired pneumonia; POA, present on admission.

^a^ Other race includes American Indian or Alaskan Native, Native Hawaiian or other Pacific Islander, and unknown.

In a subgroup analysis, we identified patients who received a procalcitonin (PCT) test on day 1 and whose PCT results were available in our dataset, as not all hospitals contribute laboratory results to Premier. We categorized PCT results as positive (≥0.25 ng/mL), indicating increased likelihood of bacterial infection, or negative (<0.25 ng/mL).^42^ We compared the primary outcome by exposure and PCT groups with χ^2^ tests and replicated the covariate-adjusted outcomes model with inclusion of PCT result (positive, negative) and an interaction term for PCT result and CAP antibiotic treatment. *P* values were 2-sided, and statistical significance was set at *P* < .05. All statistical analyses were completed in SAS software version 9.4. Data were analyzed from April to October 2024.

## Results

Our cohort included 520 405 patients with COVID-19 (median [IQR] age, 66 [53-78] years; 266 186 [51.2%] male) from 1053 hospitals, including 92 708 Black patients (17.8%), 63 619 Hispanic patients (12.2%), and 304 649 White patients (58.5%); 279 656 patients (53.7%) had Medicare insurance (Table 1). A total of 160 482 patients (30.8%) were treated with a CAP antibiotic on day 1. Patients had a median (IQR) of 3 (1-4) Elixhauser comorbidities; individual comorbidities are presented in eTable 7 in Supplement 2. For patients treated with and without a CAP antibiotic on day 1, 67.3% and 14.1%, respectively, received additional CAP antibiotic treatment during their hospitalization, for an overall median (IQR) of 3 (2-5) DOT. The median (IQR) length of stay was 4 (2-6) days, and 39 266 patients (7.9%) were readmitted to the same hospital within 30 days. Overall, 95 055 patients (18.3%) deteriorated and 22 355 patients (4.3%) died during their hospitalization.

The only patient characteristic that was meaningfully different was race and ethnicity (ASD, 10.1%): treated patients were more likely to be Hispanic or other race or ethnicity (Table 1). The use of several diagnostic tests and COVID-19 treatments on day 1 was substantially higher in the CAP treatment group, including blood culture, lactate, procalcitonin, remdesivir, and systemic steroids. Hospital characteristics varied across groups, with antibiotic-treated patients being more likely to be admitted to hospitals with fewer than 400 beds, hospitals in the South and West, rural hospitals, and nonteaching hospitals.

The matched cohort included 113 506 pairs (70.7%; *C* statistic, 0.821). After propensity matching, there was greater covariate balance between the groups, with only blood culture order having an ASD greater than 1% (ASD, 2.3%) (Table 1). The propensity score distribution by group and the propensity score model are available in the eFigure and eTable 8 in Supplement 2, respectively.

The primary composite outcome did not reach our predefined level of clinical significance between groups (ASD, 4.1%), with deterioration or in-hospital mortality occurring in 33 350 patients treated with CAP (20.8%) on day 1 vs 66 287 patients in the unexposed group (18.4%) (Table 2). Secondary and safety outcomes also were not meaningfully different between the exposure groups (Table 2).

**Table 2.** Outcomes for Patients Hospitalized With COVID-19 Treated With and Without CAP Antibiotics

Outcome	Patients, No. (%)			ASD, %
	All (n = 520 405)	No day 1 antibiotic (n = 359 923)	CAP antibiotic on day 1 (n = 160 482)	
Primary composite outcome	99 637 (19.1)	66 287 (18.4)	33 350 (20.8)	4.1
In-hospital mortality	22 355 (4.3)	13 642 (3.8)	8713 (5.4)	7.8
Deterioration	95 055 (18.3)	63 326 (17.6)	31 729 (19.8)	5.6
High-flow oxygen	7448 (1.4)	4949 (1.4)	2499 (1.6)	1.5
ICU admission	28 535 (5.5)	17 803 (5.0)	10 732 (6.7)	7.4
Intermediate care	58 593 (11.3)	39 824 (11.1)	18 769 (11.7)	2.0
Invasive mechanical ventilation	19 732 (3.8)	11 845 (3.3)	7887 (4.9)	8.2
Noninvasive ventilation	32 926 (6.3)	20 623 (5.7)	12 303 (7.7)	7.8
Vasopressor	12 495 (2.4)	7760 (2.2)	4735 (3.0)	5.0
Secondary outcomes				
30-d Readmission	39 266 (7.9)	28 182 (8.1)	11 084 (7.3)	3.1
Length of stay, median (IQR), d	4 (2-6)	3 (2-6)	4 (2-7)	8.5
Safety outcomes				
Allergic reaction	3227 (0.6)	2323 (0.7)	904 (0.6)	1.1
*Clostridioides difficile* infection	468 (0.1)	332 (0.1)	136 (0.1)	0.3
Kidney organ failure (not POA)	23 510 (4.5)	16 739 (4.7)	6771 (4.2)	2.1

Abbreviations: ASD, absolute standardized difference; CAP, community-acquired pneumonia; ICU, intensive care unit; POA, present on admission.

### Multivariable Analysis

The unadjusted model found patients with COVID-19 treated with CAP antibiotics on day 1 had higher odds of the primary composite outcome compared to the unexposed group (odds ratio [OR], 1.33 [95% CI, 1.31-1.35]; *P* < .001) (Table 3). We also found higher odds of poor clinical outcomes associated with receipt of CAP antibiotics using propensity score models (propensity-matched OR, 1.03 [95% CI, 1.01-1.05]; *P* = .003; IPTW OR, 1.03 [95% CI, 1.02-1.05]; *P* < .001; SMRW OR, 1.10 [95% CI, 1.08-1.12]; *P* < .001). Sensitivity analysis involving models of deterioration and in-hospital mortality as separate outcomes had similar results, with mortality models having higher ORs (eTable 6 in Supplement 2).

**Table 3.** Deterioration or In-Hospital Mortality for Community-Acquired Pneumonia Antibiotic vs No Antibiotic Treatment Day 1 of Admission in Patients Hospitalized for COVID-19

Model	Odds ratio (95% CI)	*P* value
Unadjusted^a^	1.33 (1.31-1.35)	<.001
Adjusted for covariates^a^	1.07 (1.05-1.09)	<.001
Adjusted for covariates and propensity score^a^	1.64 (1.41-1.90)	<.001
Propensity score matched^b^	1.03 (1.01-1.05)	.003
SMRW^a^	1.10 (1.08-1.12)	<.001
IPTW^a^	1.03 (1.02-1.05)	<.001

Abbreviations: IPTW, inverse probability treatment weighting; SMRW, standardized mortality ratio weighting.

^a^ Entire cohort: community-acquired pneumonia antibiotics on day 1: 160 482 patients; no antibiotics on day 1: 359 923 patients.

^b^ Matched cohort: 113 506 patients in both groups.

### Subgroup Analysis of Antibiotic Treatment by Procalcitonin Results

A total of 33 617 patients in the cohort had a PCT laboratory result on the day of admission. Among this subgroup, 13 768 patients (41.0%) received a CAP antibiotic on day 1. The primary composite outcome was not different by PCT result and CAP antibiotic exposure (no antibiotic vs CAP antibiotic: PCT <0.25, 3002 patients [18.0%] vs 1871 patients [18.4%]; *P* = .45; PCT ≥0.25, 920 patients [28.9%] vs 974 patients [27.1%], *P* = .11). The interaction between PCT result and CAP antibiotic treatment was not significant in a covariate-adjusted model (eTable 9 in Supplement 2).

## Discussion

In this cohort study of 520 405 patients with COVID-19 admitted to general care at 1053 hospitals, we found that initiating CAP antibiotics on day 1 of hospitalization was associated with higher odds of deterioration and in-hospital mortality. Using a rigorous target trial emulation design, these results were robust to a variety of modeling techniques and sensitivity analyses.

There is an ongoing lack of consensus on the use of antibiotics for patients with suspected viral pneumonia due to the difficulty of ruling out bacterial coinfection.^43^ To our knowledge, there have been no RCTs comparing antibiotic treatment vs placebo in adults with viral pneumonia, but retrospective studies have identified risk of adverse events and increased bacterial resistance in patients hospitalized for CAP who received antibiotics.^44,45,46,47^ A systematic review and meta-analysis reported 60.8% (95% CI, 38.6%-79.3%) of hospital-acquired bacterial infections in inpatients with COVID-19 were resistant to antibiotics, and previous antibiotic treatment was identified as a significant risk factor (OR, 2.70 [95% CI, 1.28-5.70]).^48^ Therefore, the risk of increasing antibiotic resistance at both the patient and global level due to unnecessary antibiotic prescribing needs to be assessed against the benefit of antibiotics for patients with nonsevere COVID-19.

To our knowledge, this study is the largest investigation into clinical outcomes for patients with COVID-19 treated with and without antibiotics to date. Previous research has primarily focused on in-hospital mortality^49,50,51,52,53^ and data from the first half of 2020,^16,17,49,51,52^ with the most recent study including data through June 2022.^19^ A study by Widere et al^19^ included 322 867 admissions from March 2020 to June 2022 across 66 US health systems. Using propensity matching, Widere et al^19^ found higher odds of in-hospital mortality associated with early empirical antibiotics vs no early empirical antibiotics (OR, 1.27 [95% CI, 1.23-1.33]). Important limitations of Widere et al^19^ include use of proxy measures for level of care and not adjusting for antibiotic use targeting nonpneumonia bacterial infections.

Our study also found higher odds of clinical deterioration and in-hospital mortality in propensity-matched and propensity score–weighted models with CAP antibiotic treatment on the day of admission. Although most propensity-matched pairs came from low propensity scores, ie, low likelihood of CAP antibiotic treatment, results were consistent across adjusted models. Specifically, ORs were greater than 1 in the propensity-matched model, which represents the average treatment effect among patients unlikely to be treated, and in the IPTW model, which represents the average treatment effect across the study population (approximately 70% unexposed). Alternatively, the SMRW model reweighted the untreated group to be representative of the treated group and had higher odds of having a poor outcome if treated with antibiotics.

Given the known low rates (approximately 5%) of confirmed bacterial coinfections in COVID-19, it is unsurprising that our study and others have found no improvement in clinical outcomes for patients with COVID-19 treated with empirical antibiotics.^1,2^ Several studies have proposed that gut dysbiosis is associated with COVID-19 severity, offering a potential causal mechanism for the worse outcomes associated with antibiotic treatment in our study.^17,54,55,56,57^ Disruption in the respiratory microbiome has also been associated with COVID-19, and studies have speculated that the gut-lung axis, which has previously been shown to modulate lung immunity, has a role in COVID-19 severity.^56,58,59,60,61^

Further, a meta-analysis of RCTs comparing antibiotic therapy durations found increased odds of adverse events for each additional day of antibiotic therapy.^62^ In our study, *C difficile* infection rates may have been ameliorated by low horizontal transmission because of high personal protective equipment use for COVID-19 and short median (3 DOT) therapy durations. The most common CAP antibiotic agents, ceftriaxone and azithromycin, have lower risk for acute kidney injury, which, along with short treatment courses, may explain the lack of difference in kidney failure by exposure group.^63^ Allergic reactions were also not different by exposure, perhaps because *ICD-10* codes for identifying allergic reactions have not been validated and drug adverse events are thought to be underreported in administrative data.^64^

An additional strength of this study was a sensitivity analysis limited to patients who underwent PCT testing at the time of admission. PCT is a host response biomarker intended to indicate the likelihood of bacterial pneumonia.^65,66,67,68^ However, PCT levels may be elevated in patients with COVID-19 due to the inflammatory disease process, making the utility of PCT in distinguishing bacterial coinfections unclear.^69,70,71^ We found the interaction between an elevated PCT and day 1 CAP antibiotic treatment was not significant in a covariate-adjusted logistic regression model. These results suggest the standard PCT cutoff of 0.25 ng/mL may not be appropriate for identifying bacterial coinfection in patients with COVID-19.

### Limitations

This study has several limitations. First, although our analysis adjusted for a wide range of potential confounders through robust propensity score methods and used a target trial emulation framework, we may not have completely eliminated confounding by indication, ie, a clinical indication for treatment that influences outcomes. Second, we did not have access to physiologic data, such as respiratory rate and oxygen saturation, which would have improved our ability to adjust for differences in severity of illness at presentation. Third, we identified comorbidities via diagnoses recorded during the index hospitalization and did not have access to information about comorbidities from the outpatient setting. Fourth, we relied on administrative data to identify patients with COVID-19, which may be less accurate than had we relied on laboratory test results. Fifth, we did not register a prespecified protocol, and our primary composite outcome was modified to exclude 30-day readmissions based on feedback obtained during peer review.

## Conclusions

In this large cohort study of patients hospitalized with nonsevere COVID-19, there was no clinically meaningful difference in outcomes with early antibiotic treatment targeting bacterial CAP. Given the known risks from unnecessary antibiotic treatment, antibiotic stewardship strategies for promoting appropriate antibiotic use among patients admitted with nonsevere COVID-19 are needed.

## Notes

Supplement 1. **eTable 1.** *ICD-10* Diagnosis Codes Always Requiring Antibiotics **eTable 2.** List of Immunomodulating Medications and Antibiotics

Supplement 2. **eTable 3.** Ventilation Outcome *ICD-10-PCS* Codes **eTable 4.** Vasopressor Outcome Billing Codes **eTable 5.** Primary Composite Outcome by Year **eTable 6.** Mortality and Deterioration Models **eTable 7.** Individual Elixhauser Comorbidities **eFigure.** Propensity Score Distribution **eTable 8.** Propensity Score Model **eTable 9.** Procalcitonin Subgroup Analysis Model

Supplement 3.
Data Sharing Statement
