# Supplementary material for: Antibiotic Treatment in Patients Hospitalized for Nonsevere COVID-19
Source: JAMA Netw Open. 2025 May 19;8(5):e2511499. doi: 10.1001/jamanetworkopen.2025.11499 (PMC12090033; doi:10.1001/jamanetworkopen.2025.11499)
Supplement: Supplement 2. — eTable 3. Ventilation Outcome ICD-10-PCS Codes eTable 4. Vasopressor Outcome Billing Codes eTable 5. Primary Composite Outcome by Year eTable 6. Mortality and Deterioration Models eTable 7. Individual Elixhauser Comorbidities eFigure. Propensity Score Distribution eTable 8. Propensity Score Model eTable 9. Procalcitonin Subgroup Analysis Model [file jamanetwopen-e2511499-s002.pdf]

Supplemental Online Content

Pulia MS, Griffin M, Schwei R, et al. Antibiotic treatment in patients hospitalized for nonsevere COVID-19. *JAMA Netw Ope*n. 2025;8(5):e2511499. doi:10.1001/jamanetworkopen.2025.11499

eTable 3. Ventilation Outcome *ICD-10-PCS* Codes
eTable 4. Vasopressor Outcome Billing Codes
eTable 5. Primary Composite Outcome by Year
eTable 6. Mortality and Deterioration Models
eTable 7. Individual Elixhauser Comorbidities
eFigure. Propensity Score Distribution
eTable 8. Propensity Score Model
eTable 9. Procalcitonin Subgroup Analysis Model

This supplemental material has been provided by the authors to give readers additional information about their work.

© 2025 Pulia MS et al. *JAMA Network Open*.

eTable 3. Ventilation outcome ICD-10-PCS procedure codes

Outcome	ICD-10-PCS Procedure Code	ICD-10-PCS Description
NIV	5A09357	Assistance with Respiratory Ventilation, Less than 24 Consecutive Hours, Continuous Positive Airway Pressure
NIV	5A09457	Assistance with Respiratory Ventilation, 24-96 Consecutive Hours, Continuous Positive Airway Pressure
NIV	5A09557	Assistance with Respiratory Ventilation, Greater than 96 Consecutive Hours, Continuous Positive Airway Pressure
IMV	0BH18EZ	Insertion of Endotracheal Airway into Trachea, Via Natural or Artificial Opening Endoscopic
IMV	0BH17EZ	Insertion of Endotracheal Airway into Trachea, Via Natural or Artificial Opening
IMV	5A1935Z	Respiratory Ventilation, Less than 24 Consecutive Hours
IMV	5A1945Z	Respiratory Ventilation, 24-96 Consecutive Hours
IMV	5A1955Z	Respiratory Ventilation, Greater than 96 Consecutive Hours

NIV: Non-invasive ventilation; IMV: Invasive mechanical ventilation

eTable 4. Vasopressor outcome billing codes

Medication	Billing Code Description
Dopamine	DOPAMINE IVPB 200MG 250ML
Dopamine	DOPAMINE IVPB 400MG 250ML
Dopamine	DOPAMINE IVPB 400MG 500ML
Dopamine	DOPAMINE IVPB 800MG 250ML
Dopamine	DOPAMINE IVPB 800MG 500ML
Dopamine	DOPAMINE IVPB 1600MG 500ML
Epinephrine	EPINEPHRINE 4MG/250ML
Epinephrine	EPINEPHRINE 8MG/250ML
Norepinephrine	NOREPINEPHRINE 4MG/250ML
Norepinephrine	NOREPINEPHRINE 8MG/250ML
Norepinephrine	NOREPINEPHRINE 16MG/250ML
Phenylephrine	PHENYLEPH IVPB 20MG/250ML
Phenylephrine	PHENYLEPH IVPB 30MG/250ML
Phenylephrine	PHENYLEPH IVPB 40MG/250ML
Vasopressin	VASOPRESSIN, PITRESSIN AMP 20U/ML 0.5ML
Vasopressin	VASOPRESSIN, PITRESSIN AMP 20U/ML 1ML
Vasopressin	VASOPRESSIN, PITRESSIN AMP 20U/ML 5ML

© 2025 Pulia MS et al. *JAMA Network Open*.

eTable 5. Annual outcomes for patients hospitalized with COVID-19 treated with and without community-acquired pneumonia (CAP) antibiotics on day 1 of admission

	All Patients<br>*n = 520,405*	No Antibiotic Day 1<br>*n = 359,923*	CAP Antibiotic Day 1<br>*n = 160,482*
Primary Composite Outcome			
2020	28,097 (21.9%)	15,150 (21.1%)	12,947 (22.9%)
2021	43,593 (22.2%)	29,357 (21.9%)	14,236 (23.0%)
2022	21,158 (15.2%)	16,462 (14.9%)	4,696 (16.1%)
2023	6,789 (12.1%)	5,318 (12.2%)	1,471 (11.5%)
In-Hospital Mortality			
2020	7,890 (6.1%)	3,959 (5.5%)	3,931 (6.9%)
2021	10,225 (5.2%)	6,604 (4.9%)	3,621 (5.8%)
2022	3,556 (2.6%)	2,581 (2.3%)	975 (3.4%)
2023	684 (1.2%)	498 (1.1%)	186 (1.5%)
Deterioration			
2020	26,047 (20.3%)	14,007 (19.5%)	12,040 (21.3%)
2021	42,396 (21.6%)	28,531 (21.2%)	13,865 (22.4%)
2022	20,129 (14.5%)	15,702 (14.3%)	4,427 (15.2%)
2023	6,483 (11.5%)	5,086 (11.7%)	1,397 (11.0%)

eTable 6. Model outcomes for CAP antibiotic (n = 160,482) vs. no antibiotic treatment (n = 359,923) day 1 of admission in patients hospitalized for COVID-19

In-Hospital Mortality		Odds Ratio (95% CI)	P value
	Unadjusted	1.59 (1.54, 1.63)	<0.001
	Adjusted for covariates	1.17 (1.12, 1.21)	<0.001
	Adjusted for covariates and propensity score	0.95 (0.76, 1.20)	0.67
	Propensity score matched	1.09 (1.05, 1.13)	<0.001
	SMRW	1.21 (1.16, 1.25)	<0.001
	IPTW	1.17 (1.13, 1.21)	<0.001
Deterioration			
	Unadjusted	1.31 (1.29, 1.34)	<0.001
	Adjusted for covariates	1.07 (1.05, 1.09)	<0.001
	Adjusted for covariates and propensity score	1.72 (1.48, 2.00)	<0.001
	Propensity score matched	1.03 (1.01, 1.05)	0.006
	SMRW	1.10 (1.08, 1.12)	<0.001
	IPTW	1.03 (1.02, 1.05)	<0.001

CAP: Community-acquired pneumonia; SMRW: Standardized mortality ratio weighting; IPTW: Inverse probability treatment weighting

© 2025 Pulia MS et al. *JAMA Network Open*.

eTable 7. Elixhauser comorbidities of patients hospitalized with COVID-19 by CAP antibiotic exposure

		All Cohort			Matched Cohort		
	All Patients	No Antibiotic Day 1	CAP Antibiotic Day 1	ASD	No Antibiotic Day 1	CAP Antibiotic Day 1	ASD
	n = 520,405	*n = 359,923*	*n = 160,482*		*n = 113,506*	*n = 113,506*	
Comorbidities							
Cerebrovascular disease	3.2%	3.6%	2.5%	6.2%	2.9%	2.9%	0.1%
Chronic pulmonary disease	17.5%	18.0%	16.3%	4.5%	17.3%	17.2%	0.4%
Coagulopathy	6.9%	6.9%	7.1%	0.9%	7.2%	7.2%	0.1%
Dementia	9.8%	10.2%	9.0%	4.2%	10.1%	9.9%	0.8%
Depression	11.2%	11.8%	9.8%	6.5%	10.7%	10.6%	0.3%
Diabetes with chronic complications	22.3%	22.1%	22.7%	1.5%	22.8%	22.8%	0.0%
Diabetes without chronic complications	12.8%	12.6%	13.4%	2.3%	13.0%	13.1%	0.1%
Drug abuse	2.3%	2.6%	1.6%	6.9%	1.8%	1.8%	0.2%
Heart failure	12.4%	13.1%	10.9%	6.7%	12.1%	11.9%	0.4%
Hypertension, complicated	24.6%	25.7%	22.2%	8.2%	24.2%	24.1%	0.3%
Hypertension, uncomplicated	40.8%	40.3%	41.9%	3.2%	41.2%	41.3%	0.1%
Hypothyroidism	14.2%	14.5%	13.5%	2.8%	14.2%	14.1%	0.4%
Liver disease, mild	3.9%	3.8%	4.1%	1.4%	4.0%	4.0%	0.2%
Liver disease, moderate to severe	0.6%	0.6%	0.7%	0.3%	0.7%	0.7%	0.2%
Metastatic cancer	1.1%	1.2%	0.9%	3.5%	1.1%	1.0%	0.5%
Neurological disorders affecting movement	2.3%	2.3%	2.2%	1.2%	2.3%	2.3%	0.1%
Obesity	24.1%	23.1%	26.2%	7.1%	24.8%	25.0%	0.3%
Other neurological disorders	6.6%	6.7%	6.5%	1.1%	7.2%	7.0%	0.7%
Other thyroid disorders	1.5%	1.6%	1.3%	2.7%	1.4%	1.3%	0.2%
Paralysis	2.7%	2.9%	2.1%	5.4%	2.4%	2.4%	0.4%
Peptic ulcer disease with bleeding	0.4%	0.4%	0.3%	1.4%	0.4%	0.3%	0.5%
Peripheral vascular disease	4.1%	4.3%	3.4%	4.7%	3.8%	3.8%	0.3%
Psychoses	3.2%	3.5%	2.6%	5.1%	3.0%	2.9%	0.2%
Pulmonary circulation disease	2.3%	2.4%	2.0%	2.3%	2.2%	2.2%	0.0%
Renal failure, moderate	10.6%	10.7%	10.4%	0.8%	10.9%	10.9%	0.1%
Renal failure, severe	5.5%	6.0%	4.3%	7.5%	4.9%	4.9%	0.1%
Seizures and epilepsy	2.6%	2.7%	2.3%	2.7%	2.6%	2.5%	0.4%
Solid tumor without metastasis, in situ	0.0%	0.0%	0.0%	0.6%	0.0%	0.0%	0.2%
Solid tumor without metastasis, malignant	1.8%	1.9%	1.6%	1.7%	1.8%	1.8%	0.3%
Valvular disease	4.7%	5.1%	3.8%	6.1%	4.3%	4.3%	0.1%
Weight Loss	4.2%	4.4%	4.0%	1.9%	4.4%	4.2%	0.7%

ASD: Absolute standardized difference; CAP: Community-acquired pneumonia

© 2025 Pulia MS et al. *JAMA Network Open*.

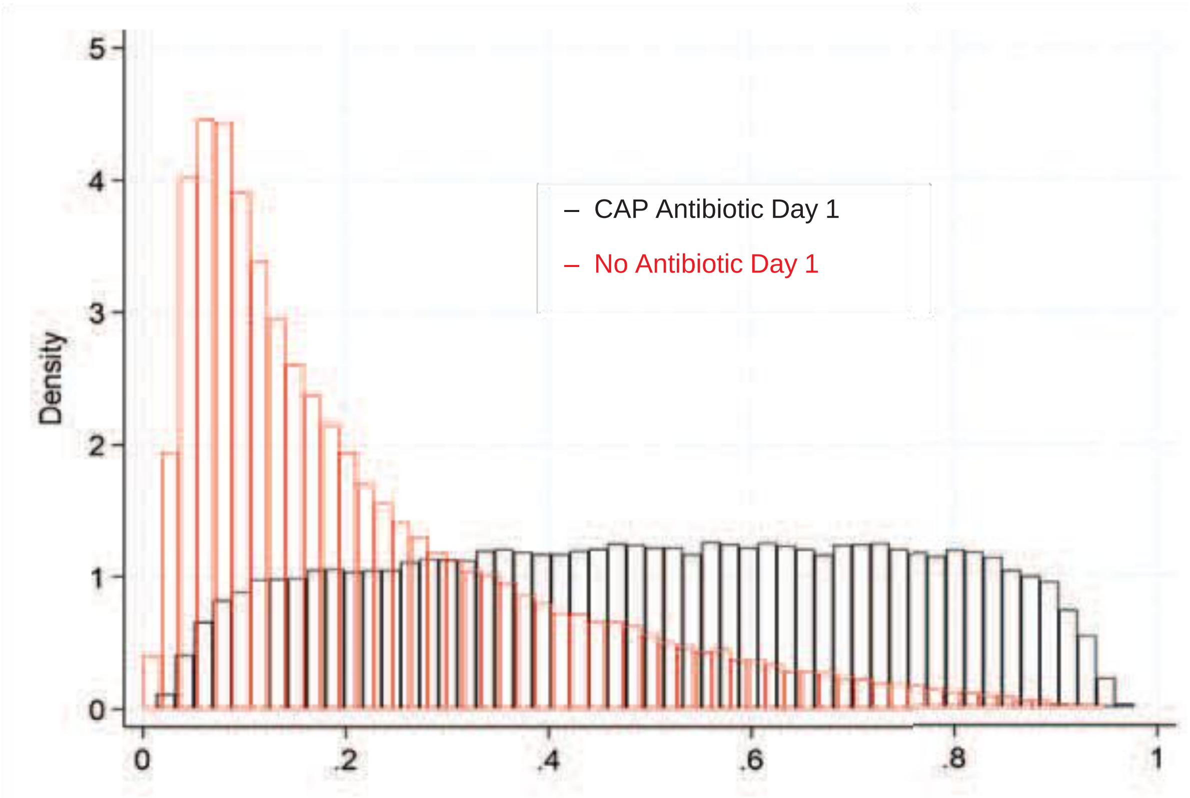

eFigure 1. Propensity score distribution for patients hospitalized with COVID-19 treated with and without community-acquired pneumonia (CAP) antibiotics on day 1 of admission

© 2025 Pulia MS et al. *JAMA Network Open*.

eTable 8. Propensity score model to predict receipt of community-acquired pneumonia antibiotic on day 1 of admission

Variable		Odds Ratio (95% CI)	p
(Intercept)		0.4 (0.34, 0.49)	<0.001
Age			
	18-20	0.66 (0.59, 0.73)	<0.001
	21-30	0.71 (0.67, 0.75)	<0.001
	31-40	0.76 (0.73, 0.80)	<0.001
	41-50	0.78 (0.75, 0.82)	<0.001
	51-60	0.81 (0.78, 0.84)	<0.001
	61-70	0.82 (0.80, 0.85)	<0.001
	71-88	0.88 (0.85, 0.90)	<0.001
	89+	Ref	Ref
Gender			
	Male	0.99 (0.98, 1.00)	0.16
	Female	Ref	Ref
Race/Ethnicity			
	Black	1.01 (0.99, 1.03)	0.30
	Hispanic	1.11 (1.08, 1.14)	<0.001
	Other	1.06 (1.03, 1.09)	<0.001
	White	Ref	Ref
Insurance			
	Medicaid	1.03 (1.01, 1.05)	0.25
	Private	1.04 (1.00, 1.07)	0.02
	Uninsured/Other/Unknown	0.98 (0.96, 1.01)	0.02
	Medicare	Ref	Ref
Comorbidities			
	Cerebrovascular disease	0.88 (0.84, 0.93)	<0.001
	Chronic pulmonary disease	0.96 (0.95, 0.98)	<0.001
	Coagulopathy	1.07 (1.02, 1.12)	0.003
	Dementia	0.93 (0.91, 0.96)	<0.001
	Depression	0.92 (0.90, 0.95)	<0.001
	Diabetes with chronic complications	1.01 (0.99, 1.03)	0.17
	Diabetes without chronic complications	0.99 (0.97, 1.01)	0.50
	Drug abuse	0.85 (0.81, 0.90)	<0.001
	Heart failure	0.99 (0.96, 1.02)	0.41
	Hypertension, complicated	0.92 (0.89, 0.94)	<0.001
	Hypertension, uncomplicated	0.95 (0.94, 0.97)	<0.001
	Hypothyroidism	0.96 (0.94, 0.98)	<0.001
	Liver disease, mild	1.06 (1.02, 1.10)	0.002
	Liver disease, moderate to severe	1.59 (1.45, 1.74)	<0.001
	Metastatic cancer	0.93 (0.87, 1.00)	0.04
	Neurological disorders affecting movement	1.00 (0.95, 1.04)	0.84
	Obesity	1.01 (1.00, 1.03)	0.12
	Other neurological disorders	0.95 (0.91, 0.99)	0.02
	Other thyroid disorders	0.90 (0.84, 0.95)	<0.001

© 2025 Pulia MS et al. *JAMA Network Open*.

Paralysis	0.96 (0.91, 1.02)	0.20
Peptic ulcer disease with bleeding	0.96 (0.85, 1.08)	0.50
Peripheral vascular disease	0.95 (0.92, 0.99)	0.007
Psychoses	0.89 (0.85, 0.92)	<0.001
Pulmonary circulation disease	1.09 (1.04, 1.15)	<0.001
Renal failure, moderate	1.05 (1.02, 1.08)	0.002
Renal failure, severe	0.82 (0.79, 0.85)	<0.001
Seizures and epilepsy	0.94 (0.90, 0.98)	0.006
Solid tumor without metastasis, in situ	0.64 (0.36, 1.15)	0.14
Solid tumor without metastasis, malignant	1.04 (0.99, 1.10)	0.13
Valvular disease	0.93 (0.90, 0.97)	<0.001
Weight Loss	1.16 (1.12, 1.20)	<0.001
Organ Failure		
Acidosis	1.12 (1.09, 1.16)	<0.001
Cardiovascular shock	0.91 (0.88, 0.93)	<0.001
Hematological	0.96 (0.92, 1.01)	0.09
Hepatic	0.90 (0.83, 0.99)	0.02
Neurologic	1.10 (1.06, 1.14)	<0.001
Renal	1.07 (1.05, 1.09)	<0.001
Respiratory	1.20 (1.15, 1.25)	<0.001
Early Treatment		
Blood culture	4.19 (4.12, 4.26)	<0.001
Lactate	1.26 (1.24, 1.28)	<0.001
Monoclonal antibodies	0.83 (0.77, 0.89)	<0.001
Procalcitonin	1.68 (1.65, 1.71)	<0.001
Remdesivir	1.22 (1.20, 1.24)	<0.001
Systemic steroid	1.81 (1.78, 1.85)	<0.001
Venous Thromboembolism Present on Admission		
Deep vein thrombosis	0.95 (0.88, 1.01)	0.1
Pulmonary embolism	1.09 (1.04, 1.15)	<0.001
Admission Quarter		
2020-Q3	0.57 (0.55, 0.60)	<0.001
2020-Q4	0.44 (0.43, 0.46)	<0.001
2021-Q1	0.35 (0.34, 0.36)	<0.001
2021-Q2	0.31 (0.30, 0.33)	<0.001
2021-Q3	0.29 (0.28, 0.30)	<0.001
2021-Q4	0.25 (0.24, 0.26)	<0.001
2022-Q1	0.24 (0.23, 0.25)	<0.001
2022-Q2	0.21 (0.20, 0.22)	<0.001
2022-Q3	0.21 (0.20, 0.22)	<0.001
2022-Q4	0.26 (0.25, 0.27)	<0.001
2023-Q1	0.27 (0.25, 0.28)	<0.001
2023-Q2	0.30 (0.28, 0.32)	<0.001
2023-Q3	0.27 (0.26, 0.29)	<0.001
2023-Q4	0.27 (0.26, 0.29)	<0.001

© 2025 Pulia MS et al. *JAMA Network Open*.

2020-Q2	Ref	Ref
Hospital Bed Size		
<200	1.25 (1.05, 1.48)	0.01
200-399	1.12 (0.95, 1.32)	0.18
399+	Ref	Ref
Hospital Census Region		
Midwest	0.72 (0.61, 0.85)	<0.001
Northeast	0.59 (0.49, 0.72)	<0.001
South	1.08 (0.93, 1.27)	0.31
West	Ref	Ref
Hospital Rurality		
Rural	1.16 (1.02, 1.33)	0.03
Urban	Ref	Ref
Teaching Hospital		
No	1.23 (1.07, 1.42)	0.003
Yes	Ref	Ref

© 2025 Pulia MS et al. *JAMA Network Open*.

eTable 9. Logistic regression model to predict primary composite outcome (deterioration or in-hospital mortality) among subgroup of patients hospitalized for COVID-19 with procalcitonin (PCT) lab result

Variable		OR (95% CI)	p
(Intercept)		0.2 (0.11, 0.38)	<0.001
CAP Antibiotic Day 1		1.08 (1, 1.17)	0.06
PCT 2: 0.25 ng/mL		1.49 (1.34, 1.66)	<0.001
CAP Antibiotic Day 1 * PCT 2: 0.25 ng/mL		0.97 (0.84, 1.12)	0.70
Age			
	18-20	0.48 (0.26, 0.9)	0.02
	21-30	0.57 (0.45, 0.73)	<0.001
	31-40	0.68 (0.56, 0.83)	<0.001
	41-50	0.76 (0.64, 0.91)	0.002
	51-60	0.83 (0.71, 0.99)	0.03
	61-70	0.88 (0.76, 1.03)	0.12
	71-88	0.88 (0.76, 1.01)	0.07
	89+	Ref	Ref
Gender			
	Male	1.18 (1.1, 1.25)	<0.001
	Female	Ref	Ref
Race/Ethnicity			
	Black	0.81 (0.73, 0.89)	<0.001
	Hispanic	1.08 (0.97, 1.2)	0.18
	Other	1.09 (0.98, 1.23)	0.13
	White	Ref	Ref
Insurance			
	Private	1.16 (1.06, 1.26)	<0.001
	Uninsured/Other/Unknown	1.05 (0.93, 1.18)	0.45
	Medicaid/Medicare	Ref	Ref
Comorbidities			
	Cerebrovascular disease	1.2 (0.86, 1.66)	0.28
	Chronic pulmonary disease	1.08 (1, 1.17)	0.06
	Coagulopathy	0.95 (0.79, 1.14)	0.60
	Dementia	0.86 (0.76, 0.98)	0.02
	Depression	1.12 (1.02, 1.23)	0.02
	Diabetes with chronic complications	1.29 (1.2, 1.4)	<0.001
	Diabetes without chronic complications	0.93 (0.85, 1.03)	0.18
	Drug abuse	0.86 (0.67, 1.11)	0.25
	Heart failure	1.8 (1.6, 2.03)	<0.001
	Hypertension, complicated	0.83 (0.74, 0.95)	0.005
	Hypertension, uncomplicated	1.05 (0.97, 1.14)	0.20
	Hypothyroidism	1.03 (0.95, 1.13)	0.46
	Liver disease, mild	1.22 (1.05, 1.43)	0.01
	Liver disease, moderate to severe	0.85 (0.53, 1.38)	0.51
	Metastatic cancer	1.2 (0.86, 1.66)	0.28
	Neurological disorders affecting movement	1.26 (1.02, 1.55)	0.03
	Obesity	1.88 (1.75, 2.02)	<0.001

© 2025 Pulia MS et al. *JAMA Network Open*.

	Other neurological disorders	0.6 (0.5, 0.73)	<0.001
	Other thyroid disorders	1.13 (0.87, 1.47)	0.37
	Paralysis	1.06 (0.81, 1.4)	0.67
	Peptic ulcer disease with bleeding	1.77 (1.04, 2.98)	0.03
	Peripheral vascular disease	1.2 (1.02, 1.41)	0.03
	Psychoses	1.23 (1.03, 1.47)	0.02
	Pulmonary circulation disease	1.35 (1.09, 1.67)	0.005
	Renal failure, moderate	1.04 (0.92, 1.18)	0.54
	Renal failure, severe	0.84 (0.7, 1)	0.05
	Seizures and epilepsy	1.23 (1.01, 1.5)	0.04
	Solid tumor without metastasis, in situ	1.08 (0.12, 9.64)	0.94
	Solid tumor without metastasis, malignant	1 (0.78, 1.29)	0.97
	Valvular disease	1.28 (1.1, 1.49)	0.002
	Weight Loss	1.83 (1.58, 2.13)	<0.001
Organ Failure			
	Acidosis	1.77 (1.58, 1.98)	<0.001
	Cardiovascular shock	3.05 (2.74, 3.39)	<0.001
	Hematological	1.64 (1.38, 1.95)	<0.001
	Hepatic	2.94 (1.98, 4.36)	<0.001
	Neurologic	3.3 (2.79, 3.91)	<0.001
	Renal	1.46 (1.34, 1.58)	<0.001
	Respiratory	3.05 (2.74, 3.39)	<0.001
Early Treatment			
	Blood culture	1.02 (0.95, 1.1)	0.50
	Lactate	1.02 (0.94, 1.11)	0.60
	Monoclonal antibodies	0.38 (0.26, 0.56)	<0.001
	Remdesivir	1.21 (1.12, 1.3)	<0.001
	Systemic steroid	1.08 (1, 1.17)	0.05
Venous Thromboembolism Present on Admission			
	Deep vein thrombosis	1.54 (1.19, 2.01)	0.001
	Pulmonary embolism	1.85 (1.53, 2.23)	<0.001
Admission Quarter			
	2020-Q3	0.9 (0.76, 1.07)	0.22
	2020-Q4	0.73 (0.64, 0.84)	<0.001
	2021-Q1	0.67 (0.57, 0.77)	<0.001
	2021-Q2	0.7 (0.58, 0.84)	<0.001
	2021-Q3	0.88 (0.76, 1.02)	0.09
	2021-Q4	0.82 (0.7, 0.96)	0.02
	2022-Q1	0.56 (0.48, 0.66)	<0.001
	2022-Q2	0.25 (0.19, 0.34)	<0.001
	2022-Q3	0.34 (0.27, 0.43)	<0.001
	2022-Q4	0.31 (0.24, 0.4)	<0.001
	2023-Q1	0.24 (0.17, 0.32)	<0.001
	2023-Q2	0.27 (0.17, 0.44)	<0.001
	2023-Q3	0.37 (0.26, 0.51)	<0.001

© 2025 Pulia MS et al. *JAMA Network Open*.

	2023-Q4	0.25 (0.18, 0.35)	<0.001
	2020-Q2	Ref	Ref
Hospital Bed Size			
	<200	0.79 (0.56, 1.11)	0.18
	200-399	0.95 (0.68, 1.34)	0.79
	399+	Ref	Ref
Hospital Census Region			
	Midwest	0.64 (0.37, 1.11)	0.11
	Northeast	0.7 (0.39, 1.24)	0.22
	South	0.63 (0.38, 1.06)	0.08
	West	Ref	Ref
Hospital Rurality			
	Rural	0.89 (0.68, 1.16)	0.40
	Urban	Ref	Ref
Teaching Hospital			
	No	0.97 (0.72, 1.29)	0.81
	Yes	Ref	Ref

CAP: Community-acquired pneumonia; PCT: Procalcitonin

© 2025 Pulia MS et al. *JAMA Network Open*.
