# Supplementary material for: Antibiotic Treatment in Patients Hospitalized for Nonsevere COVID-19
Source: JAMA Netw Open. 2025 May 19;8(5):e2511499. doi: 10.1001/jamanetworkopen.2025.11499 (PMC12090033; doi:10.1001/jamanetworkopen.2025.11499)
Supplement: Supplement 3. — Data Sharing Statement [file jamanetwopen-e2511499-s003.pdf]

Data Sharing Statement

Pulia. Antibiotic Treatment in Patients Hospitalized for Nonsevere COVID-19. *JAMA Netw Open*. Published May 19, 2025. doi:10.1001/jamanetworkopen.2025.11499

Data
Data available: No

Additional Information
Explanation for why data not available: The Premier Healthcare Database is proprietary and access requires purchase and a contract with Premier Inc.
